# Guardians in the Gut: Mechanistic Insights into a Hidden Ally Against Triple-Negative Breast Cancer

**DOI:** 10.3390/cancers17193248

**Published:** 2025-10-07

**Authors:** Kayla Jaye, Muhammad A. Alsherbiny, Dennis Chang, Chun-Guang Li, Deep Jyoti Bhuyan

**Affiliations:** 1 NICM Health Research Institute, Western Sydney University, Penrith, NSW 2751, Australia; 19255718@student.westernsydney.edu.au (K.J.); muhammad.alsherbiny@pharma.cu.edu.eg (M.A.A.); d.chang@westernsydney.edu.au (D.C.); c.li@westernsydney.edu.au (C.-G.L.); 2 Pharmacognosy Department, Faculty of Pharmacy, Cairo University, Cairo 11562, Egypt; 3 School of Science, Western Sydney University, Penrith, NSW 2751, Australia

**Keywords:** gut microbial metabolites, triple-negative breast cancer, short-chain fatty acids, natural purine nucleosides, ellagic acid derivatives, tryptophan metabolites, standard chemotherapy

## Abstract

**Simple Summary:**

Triple-negative breast cancer is one of the most aggressive types of breast cancer and has fewer treatment options than other forms. This review was written to explore an emerging area of research: the role of the gut microbiome in cancer. The gut is home to trillions of microbes that produce natural compounds, known as metabolites, which can travel through the body and affect health in many ways. We summarise what is currently known about how these metabolites may slow cancer growth, influence gene regulation through small molecules called microRNAs, and work together with chemotherapy drugs. By bringing together these findings, this review highlights new possibilities for using gut-derived molecules to improve treatment outcomes in breast cancer. The aim is to provide researchers and clinicians with an overview of potential opportunities for developing safer and more effective strategies against this difficult-to-treat disease.

**Abstract:**

The gut microbiome possesses a diverse range of biological properties that play a role in maintaining host health and preventing disease. Gut microbial metabolites, including short-chain fatty acids, natural purine nucleosides, ellagic acid derivatives, and tryptophan metabolites, have been observed to have complex and multifaceted roles in the gut and in wider body systems in the management of disease, including cancer. Triple-negative breast cancer is the most aggressive subtype of breast cancer, with restricted treatment options and poor prognoses. Recently, preclinical research has investigated the antiproliferative potential of gut microbial metabolites against this type of breast cancer with promising results. However, little is understood about the mechanisms of action and molecular pathways driving this antiproliferative potential. Understanding the complex mechanisms of action of gut microbial metabolites on triple-negative breast cancer will be instrumental in the investigation of the combined administration with standard chemotherapeutic drugs. To date, there is a paucity of research studies investigating the potential synergistic interactions between gut microbial metabolites and standard chemotherapeutic drugs. The identification of synergistic potential between these compounds may provide alternate and more effective therapeutic options in the treatment and management of triple-negative breast cancer. Further investigation into the mechanistic action of gut microbial metabolites against this breast cancer subtype may support the administration of more cost-effective treatment options for breast cancer, with an aim to reduce side effects associated with standard treatments. Additionally, future research will aim to identify more potent metabolite–drug combinations in the mitigation of triple-negative breast cancer progression and metastasis.

## 1. Introduction

**Figure 1:**
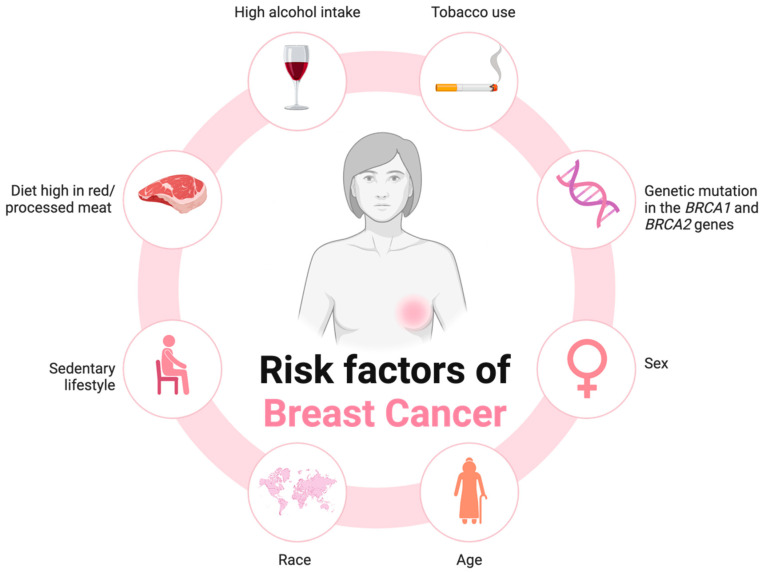
A diagrammatic representation of the most prevalent lifestyle, environmental, and genetic risk factors associated with the onset of breast cancer.

According to the World Health Organisation (WHO), breast cancer is the second most commonly occurring cancer type globally, with 2.3 million cases in 2022, representing 11.6% of all cancer diagnoses [1]. However, it is the most commonly diagnosed cancer type and the leading cause of cancer death in women on a global scale [1]. This global burden of female breast cancer incidence and mortality is projected to increase with the adoption of various lifestyle behaviours that contribute to overall breast cancer risk [2]. These risk factors include dietary factors, including a diet high in fat, red and processed meat consumption, and low fibre intake, as well as lack of physical activity, tobacco use, and alcohol intake (Figure 1) [3]. Additional risk factors for breast cancer can include genetics, race, and age, as exemplified by the genetic mutations observed in the *BRCA1* (breast cancer 1) and *BRCA2* (breast cancer 2) genes (Figure 1) [4]. Genetic risk factors, whilst an important consideration, only account for roughly 10% of all breast cancer diagnoses [3]. Despite this, as a potential heterogeneous disease, various molecular changes occur to lead to different subtypes of breast cancer, including HER2-positive, Luminal A, Luminal B, and triple-negative breast cancer (TNBC) [4].

TNBC is considered the most aggressive, invasive form of breast cancer due to being difficult to treat and often having poor prognoses, and it is more likely to metastasise to other parts of the body and return after treatment [4]. The immuno-profile of TNBC is oestrogen-, progesterone-, and HER2-negative [4]. The heightened risk of metastasis associated with TNBC is a crucial factor in therapeutic decision-making, as it can potentially metastasise to diverse organ sites, including the brain, bone, liver, and lungs, culminating in metastatic heterogeneity [5]. Previous research has reported that roughly 20–30% of people with breast cancer develop metastases following diagnosis and treatment of the primary tumour, and metastatic tumours are responsible for approximately 90% of breast cancer-related deaths [5]. The TNBC subtype accounts for roughly 15–20% of all breast cancer diagnoses and is hormone-independent, which means it does not express oestrogen or progesterone receptors, as well as the HER2 gene and this lack of potential therapeutic targets contributes to the difficulties in treating this cancer type [4].

### 1.1. The Role of the Gut Microbiota in the Maintenance of Host Health

The gut microbiota consists of 100 trillion microorganisms residing in the adult human gut, including bacteria, fungi, protozoa, and viruses, all playing an integral role in the overall maintenance of human health and prevention of disease [6,7,8]. The maternal microbiome profoundly influences the establishment of a robust infant immune system, thereby significantly protecting against chronic ailments in later stages of life [9]. The adult gut microbiota interrelates with various body systems and plays a vital role in the maintenance of host health and gut homeostasis, as well as in the prevention of diseases. The human intestinal microbiome is primarily composed of two bacterial phyla, *Firmicutes* and *Bacteroidetes*, representing more than 90% of the total gut microbial composition [10], followed by the *Actinobacteria* and *Verrucomicrobia* phyla [5]. In the adult gut microbiome, the primary genera of bacteria include *Clostridium*, *Bacteroides*, *Bifidobacterium*, *Faecalibacterium*, *Lactobacillus*, *Eubacterium*, *Streptococcus*, *Ruminococcus*, *Peptostreptococcus*, *Peptococcus*, and *Streptomyces*. These bacteria play a vital role in the symbiotic relationship with the host, aiding in digestion, metabolic processes, and immune responses as mutualistic communities [11]. The abundance and diversity of microorganisms vary throughout the gastrointestinal tract (GIT), with *Helicobacter pylori* as the dominant bacterial species residing in the stomach, *Firmicutes* and *Actinobacteria* the dominating phyla in the duodenum, *Lactobacilli*, *Streptococci*, and *Enterococci* dominating the jejunum, and the ileum dominated by similar bacterial species to the colon [5]. The most prevalent bacterial species in the large intestine are from the two phyla *Firmicutes* and *Bacteroidetes*, with important pathogens present in the colon, including *Escherichia coli*, *Bacteroides fragilis*, *Salmonella enterica*, *Campylobacter jejuni*, and *Vibrio cholera* [5]. The high abundance of *Bacteroides*, *Ruminococcus*, and *Prevotella* and the low abundance of the *Proteobacteria* phylum are indicative of a healthy gut microbial composition in humans [5].

The gut microbiota has been associated with the pathophysiology of complex diseases, including obesity, type 2 diabetes, gastrointestinal disorders, cancers, and neurological disorders. For example, small intestinal bacterial overgrowth (SIBO), also known as blind loop syndrome, is characterised by an abnormal proliferation of bacterial species within the small intestine, typically associated with microbial populations found in the lower gastrointestinal tract (GIT) [12]. This condition leads to the malabsorption of carbohydrates, fats, and proteins, which can cause vitamin deficiencies and malnutrition in affected individuals and can present as abdominal distension and pain, gas, and bloating [12].

Gut microbial metabolites, including tryptophan metabolites, short-chain fatty acids, and bile acid derivatives, can influence cancer progression and development, as well as anticancer immunity [13]. These molecules interact with tumour and immune cells via metabolic and epigenetic signalling pathways [13]. The multifaceted role of the gut microbiota in human health warrants further research into protective species and the widespread effects of microbial composition changes within the body.

### 1.2. The Complex Interplay of the Gut–Brain Axis

The multifaceted association between the gut microbiota and the maintenance of host health has been further explained by the gut–brain axis (GBA), which is a bidirectional connection between gut microorganisms and the brain that influences physiological functions within the host [14,15]. The gut microbiota can regulate levels of gut peptides, such as cholecystokinin and serotonin, to influence the vagal afferent pathway and regulate intestinal metabolic processes, collectively referred to as the microbiota-gut–brain axis [16]. The GBA has also been found to have an integral role in cancer cell proliferation and invasion, apoptosis, and metastasis, and some gut microbial species have been associated with relieving chemotherapeutic-related neurological symptoms [16]. Specifically, serotonin has been observed to promote TNBC progression via autocrine serotonin signalling processes [16]. The pivotal role of the GBA in both normal physiological processes and disease conditions within the human body necessitates thorough consideration in developing personalised and targeted therapeutic strategies for gut microbiota-associated disorders.

### 1.3. Gut Dysbiosis and Its Role in Breast Cancer Progression

Disturbances to the healthy gut microbial composition, a process known as gut dysbiosis, can lead to the onset of cancer. Gut dysbiosis can be induced by a variety of factors, including specific dietary factors, alcohol, smoking, antibiotic consumption, stress, and standard chemotherapeutics [11]. Dysbiosis of the gut is typically characterised by a reduced abundance of commensal bacterial species and alpha diversity, as well as an increase in abundance of pathobionts [5]. These microbial changes can lead to chronic inflammation in the gut, which perturbs the innate and adaptive immune responses within the host and promotes the risk of malignancy [5]. This increase in vulnerability to pathogens has been associated with the development of colon, pancreatic, gastric, gallbladder, esophageal, laryngeal, and breast cancer [17].

Gut microbial dysbiosis has been observed to contribute to breast cancer progression via both oestrogen-dependent and non-oestrogen-dependent mechanisms, including the production of microbial-derived metabolites, DNA changes, and immune response regulation [11]. This dysbiotic process may be an influencing factor in the metastasis of breast cancer, as it impacts a variety of risk factors associated with this metastatic progression [5]. The observed potential causative link between gut dysbiosis and breast cancer progression serves as a strong foundation for future research into therapeutic strategies and targeted approaches. Specific gut microbial patterns and diversity can serve as biomarkers in the diagnosis and prognosis of breast cancer, with an evident variation in gut microbial compositions between participants with breast cancer and healthy individuals [10].

One meta-analysis systematic review explored gut microbial modification from various breast cancer treatments and the impact of gut microbial patterns on participants with breast cancer receiving the same treatment, to support the use of personalised medicine to improve prognosis [10]. This review observed a significant increase in the *Methylobacterium radiotolerans* species in breast tumour tissue in comparison to healthy breast tissue, as well as a low overall intestinal microbial diversity, reduced abundance of *Firmicutes* and *Bacteroidetes*, increased *Proteobacteria*, *Actinobacteria*, and *Verrucomicrobia*, and decreased abundance of *Faecalibacterium prausnitzii* in breast cancer patients compared to healthy controls [10], and this reduction in *Faecalibacterium* abundance was also observed in a second study [18]. The gut dysbiosis induced by the onset of breast cancer was observed in one preliminary study that found that women in the early stages of breast cancer had a reduced microbial diversity in comparison to healthy women [19]. Specifically, the study found that people with breast cancer had a reduced abundance of *Bifidobacterium*, *Coprococcus*, *Odoribacter*, and *Butyricimonas*, all of which are *Bacteroidetes* species, as well as an increased abundance of two *Firmicutes* species, *Clostridium* cluster IV and *Clostridium* cluster XIVA [19]. Another study examined bacterial DNA extracted from the faecal samples of people with breast cancer using 16S rRNA sequencing and found that bacterial phyla differed according to clinical stages of breast cancer, with specific changes observed in the abundance of *Blautia* and *Bifidobacterium* [20]. Another study observed that the actual metabolic pathways of the gut microbiota differed between a benign and malignant breast tumour group, implicating the role of the gut microbiota in prognosis and progression between the two tumour types [21]. For example, the study identified an increase in lipopolysaccharide biosynthesis in the malignant tumour group compared to the benign tumour group, as well as an increase in sporulation in participants with benign tumour [21]. The increase in sporulation saw the developmental change in vegetative cells into metabolically inactive and highly resistant cells. This study also noted that participants with malignant breast tumours had an increased level of the *Citrobacter* species, whereas participants with benign breast tumours had elevated levels of *Faecalibacterium*, *Clostridium*, *Arcanobacterium*, *Lachnospira*, *Romboutsia*, *Xylophilus*, *Fusicatenibacter*, and *Erysipelotrichaceae* bacterial species [21]. Further, an ongoing clinical trial (NCT03885648) is undertaking metagenomic and metabolomic studies in women diagnosed with stage I or II breast cancer to ascertain bacterial, viral, fungal, and archaea populations in faecal and breast tissue samples [22]. This will serve as the first study to assess the combined contribution of these microbial species in the risk of breast cancer, which can provide a new understanding of new therapeutic interventions in treating this cancer type [22].

The faecal microbiome of participants with newly diagnosed breast cancer has also been observed to differ in diversity and composition in comparison to healthy controls, in which breast cancer patients were found to have greater levels of urinary estrogens, lower microbial diversity, and altered faecal microbiome composition [23]. Additionally, these participants had an increased abundance of *Clostridiaceae*, *Ruminococcaceae*, and *Faecalibacterium*, and a decreased abundance of *Lachnospiraceae* and *Dorea* species in their faecal microbiome [23]. This further supported the potential use of microbial patterns as diagnostic tools in breast cancer and could also serve as novel targets for new treatment approaches. However, the current lack of human clinical trials limits the ability to validate potential novel treatment approaches. A large-scale study of the participants with breast cancer to analyse their gut microbial composition and associated metabolome, in comparison to healthy controls, would aid in identifying new diagnostic and prognostic biomarkers for breast cancer [11].

Specific standard chemotherapeutic drugs, including cyclophosphamide (CTX) and 5-fluorouracil, are toxic to gut microbial species and either act directly on the microbiome to impact the gut microbial composition or induce immune responses to target the microbial species [24]. A pilot longitudinal study investigated the gut microbiome, including alpha diversity and bacterial species abundance and changes in participants with breast cancer in the first year of treatment following chemotherapy, no chemotherapy, adjuvant chemotherapy, and neoadjuvant chemotherapy, in which all chemotherapy treatments were found to impact the gut microbial profiles in the participants [25]. The study observed notable changes in *Bacteroidetes*, *Verrucomicrobia*, and *Firmicutes* taxa abundance amongst the different groups, as well as variation in alpha diversity abundance, and these notable changes were observed to differ by treatment in the study, such as between neoadjuvant and adjuvant chemotherapies [25]. The distinguishable variations between the gut microbiota of participants with breast cancer and healthy controls and between participants undergoing different treatment types can serve as biomarkers for disease onset and aid in developing gut microbiota-based interventions and therapeutics.

The aim of the present review is to collate existing research on the action of specific gut microbial metabolite groups on triple-negative breast cancer, as well as emphasise the emerging role of miRNA expression on both the inhibition and initiation of this breast cancer subtype. The review also aims to highlight the future potential of adjuvant therapies in the treatment of this cancer subtype via the combination of gut metabolites and standard chemotherapeutics. The novelty of this review stems from its focus on bridging mechanistic insights into gut microbial metabolites with potential therapeutic applications in triple-negative breast cancer, providing a foundation for future translational research.

## 2. Methods

### Search Strategy

The articles for the narrative review were sourced from PubMed, Scopus, Embase, and Google Scholar. Articles were searched by using the keywords “gut microbiota”, “gut microbiome”, “gastrointestinal microbiome”, “gut metabolite”, “gut microbial metabolite”, “gut microorganism”, “gut microbe”, “butyrate”, “sodium butyrate”, “Na butyrate”, “butyric acid”, “inosine”, “natural purine nucleoside”, “urolithin A”, “urolithin B”, “acetate”, “magnesium acetate tetrahydrate”, “propionate”, “sodium propionate”, “postbiotic”, “prebiotic”, “probiotic”, “tryptophan”, “indole”, “soluble fiber”, “soluble fibre”, “breast cancer”, “breast carcinogenesis”, “breast adenocarcinoma”, “triple-negative breast cancer”, “minimal residual disease”, “microRNA”, “miRNA”, “standard chemotherapy”, “breast neoplasm”, “breast cancer cell”, “breast cancer in vitro”, “breast cancer in vivo”, and “breast cancer stem cell”.

Following the initial literature search, each article was assessed for its relevance to the narrative review on the action of gut metabolites on breast cancer and the association with miRNA expression, based on the following criteria: title, abstract, and the key focus of the overall study in relation to the review. The period in the literature search was undefined and included all relevant articles published on the topic to date. An additional search strategy was used to search the reference lists of selected relevant articles to identify additional sources. The exclusion criteria for sources included: book chapters, conference proceedings, irrelevant outcomes in the source, restricted sources, and studies that did not directly discuss gut metabolites or breast cancer in the context of the review.

The narrative review process followed a pre-defined protocol, which aligned with published PRISMA guidelines, followed by a comprehensive search of all relevant scientific databases to identify studies that meet the inclusion or exclusion criteria. The final stage of the process involved critical appraisal of the quality of each included study, in which the evidence level of each study was adequality examined prior to inclusion. The analysis of these resources was carried out using the summarisation of the information in the articles, inclusive of, but not limited to, the purpose of the research study and central conclusions drawn from the studies.

## 3. Results

### 3.1. Gut Microbial Metabolites and Breast Cancer

**Figure 2:**
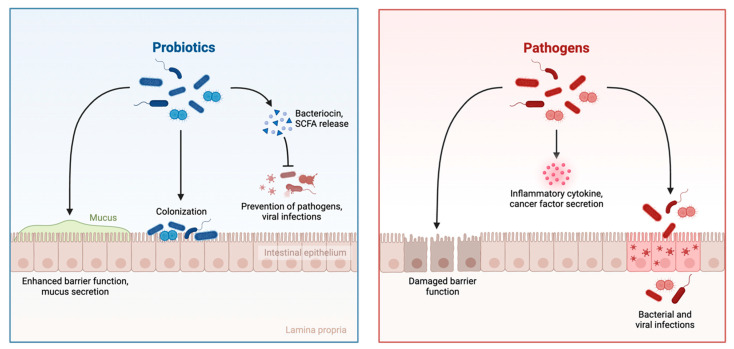
A diagrammatic overview of the effect of probiotic and pathogenic gut microbial species on host intestinal health.

The potential use of gut microbial metabolites in the treatment and management of breast cancer and in the alleviation of symptoms associated with standard chemotherapeutics has been an evolving area of research in recent years. These metabolites are produced as by-products via metabolic processes of pre- and probiotic species residing in the adult human gut (Figure 2).

**Figure 3:**
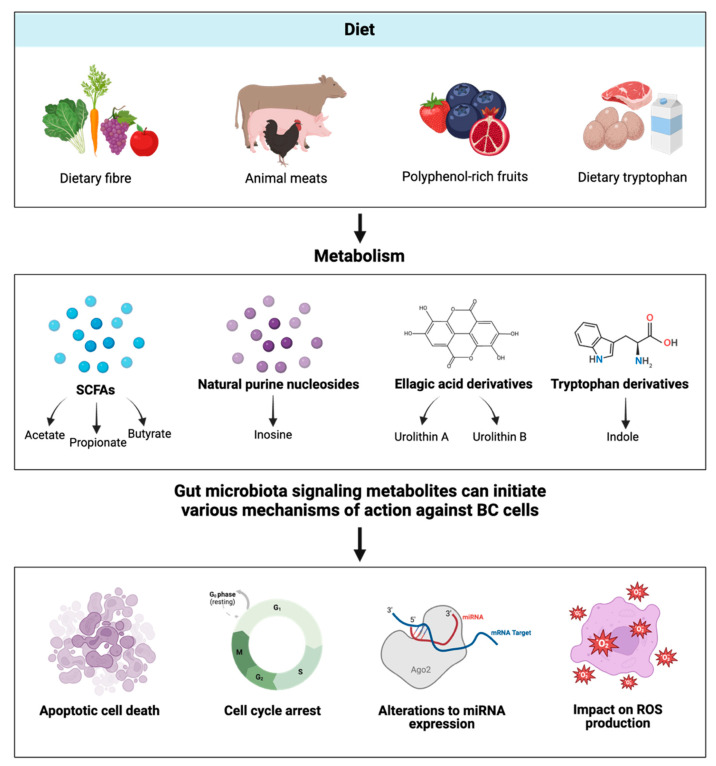
A schematic representation of the gut microbial metabolite groups and the specific food types they are derived from, as well as the mechanisms of action of these metabolites in breast cancer cells.

The most produced gut microbial metabolites are short-chain fatty acids (SCFAs, e.g., butyrate, acetate, and propionate), as well as bacteriocins, secondary bile acids, tryptophan metabolites, phenylpropanoids, ellagic acid derivatives, and natural purine nucleosides [8,14]. These compounds have been observed to possess a diverse range of biological properties in both the maintenance of host health and the inhibition of carcinogenesis and may serve as novel therapeutic approaches in the management of breast cancer (Figure 3).

#### 3.1.1. Short-Chain Fatty Acids (SCFAs)

SCFAs are the most produced gut microbial metabolites and are primarily synthesised by bacterial species in the *Firmicutes* phyla, including *Clostridium leptum*, *Eubacterium rectale*, *Eubacterium hallii*, *Anaerostipes*, and *Faecalibacterium prausitzii* [14]. The most common SCFAs are butyrate, acetate, and propionate, constituting roughly 95% of all produced SCFAs, with the remaining 5% comprising valeric, caproic, and formic acids [26]. Within the host, SCFAs possess diverse physiological properties, including altering the gut microbial composition within the GIT, regulating the physiology of the colon, participating in complex host-signalling mechanisms, and being used as energy sources for host cells [27]. Previous research has shown that SCFAs exhibit benefits for the host immune system and can reduce the overall risk of disease, including heart disease, type 2 diabetes, inflammatory disorders, obesity, and cancer. These metabolites have been observed to regulate the production of inflammatory factors responsible for the induction of the inflammatory response, such as IL-6, IL-8, TNF-α, nitric oxide, and lipopolysaccharides, and upregulate the expression of anti-inflammatory cytokines, such as IL-10 [26].

The role of SCFAs in the treatment of breast cancer has been researched preclinically, demonstrating that these metabolites exhibit promising antiproliferative potential against breast cancer cell lines in vitro. Butyrate is a key SCFA, and histone deacetylase inhibitor (HDACi) produced as a by-product of bacterial fermentation of non-digestible and fermentable carbohydrates within the gut, most primarily following the ingestion of dietary fibres [14]. Notably, HDACis have been reported to exhibit significantly lower cytotoxicity on normal cells than on cancerous cells and inhibit tumour growth and induce apoptotic cell death [4]. However, the effect of HDACis on solid tumours is not well-defined. HDACis are the first successful anticancer epigenetic therapy and have been found to positively influence the therapy of various malignancy subtypes, such as TNBC [4]. Several HDACis have been studied in clinical trials against different cancer types; however, in specific relation to TNBC, these compounds are found to be more clinically beneficial when administered as a complementary treatment or in combination with standard chemotherapeutic drugs [4].

Sodium butyrate, the sodium salt of butyrate, has been found to exhibit high potency against TNBC in both low and medium concentrations. One in vitro study assessed the antiproliferative activity of sodium butyrate on the MDA-MB-231 TNBC cell line and observed that it induced cell cycle arrest in the G_2_ growth phase via the induction of p21^Waf1^ that inhibited the activation of cyclin A- and B1-dependent kinases against this cell line [28]. Our recent research on sodium butyrate found that it was the most potent compound tested against MDA-MB-231 cell lines, inhibiting ROS production and inducing apoptotic cell death [29]. Proteomics analyses observed that sodium butyrate triggered the initiation of signal transduction processes and cellular responses to stimuli within the MCF7 cells, including regulation of the HSF1-mediated heat shock response [29]. An earlier study observed that sodium butyrate inhibited the progression of TNBC cell lines MDA-MB-231 and BT-549 and identified that this compound decreased mutant p53 (mtp53) transcription without impacting wild-type p53 (wtp53) and increased the acetylation of residues 170–200 of transcription factor YY1 (Yin Yang 1), as well as inhibiting the association of HDAC8 [30]. Importantly, sodium butyrate may serve as a promising drug candidate for the treatment of TNBC, where the HDAC8/YY1/mtp53 signals complex may be a significant treatment target [30].

Propionate is a SCFA primarily produced from carbohydrates metabolised by gut microbiota and is an agonist of two G protein-coupled receptors, GPR41 and GPR43 [31]. Propionate and its sodium salt, sodium propionate, have also been investigated preclinically for their antiproliferative properties. In a study examining the antiproliferative potential of propionate against MCF7 and MDA-MB-231 breast cancer cells, it was found to inhibit cell proliferation and invasion via activation of large tumour suppressor kinase 1, as well as the inhibition of extracellular signal-regulated kinase 1/2 in breast cancer cells overexpressing the GPR41 and GPR43 receptors [32]. Subsequent research into the sodium propionate derivative identified that this metabolite inhibited the proliferation of breast cancer cells dose-dependently and induced apoptotic cell death; however, this activity was not mediated by the two GPR41 and GPR43 receptors [31]. Notably, sodium propionate exerted this antiproliferative potential via the inhibition of JAK2/STAT3 signalling, leading to cell cycle arrest in the G_0_/G_1_ growth phase and increased ROS levels and phosphorylation of p38, which led to the induction of apoptosis in the breast cancer cells [31]. The study also examined the antitumour activity of sodium propionate in vivo, and the administration of this metabolite in nude mice bearing MCF7 and JIMT-1 cells xenograft was found to significantly inhibit tumour progression via regulation of STAT3 and p38 in the tumour tissues [31]. These findings identified the probable mechanisms of action of sodium propionate in inhibiting cell proliferation and inducing apoptosis in breast cancer cells via the inhibition of STAT3, activation of p38, and increased ROS levels [31]. Whilst sodium propionate has demonstrated promising potential as an antiproliferative agent for breast cancer, it has presented with a lower potency compared to sodium butyrate in preclinical studies [33].

Overall, SCFAs have shown promise in preclinical studies. However, more studies are paramount to understanding their in-depth molecular mechanisms of action, especially their role as localised vs. systemic treatment strategies for breast cancer. The bioavailability of SCFAs is another crucial factor to consider in administering these compounds as antiproliferative agents, as the observed effects in vitro may not translate to clinical studies if the compounds are not readily bioavailable within the human body.

#### 3.1.2. Natural Purine Nucleosides

Natural purine nucleosides are derived from primarily energy-rich foods, including animal and fish meats, yeast, and organs, such as the liver or fish milt. Adenosine, a purine primarily synthesised in the liver, has been observed in higher concentrations within the tumour microenvironment [34]. Inosine is a natural purine nucleoside formed via the metabolic conversion of adenosine by the adenosine deaminase enzyme, and the bacterial species *Bifidobacterium pseudolongum* has been observed to undergo the relevant metabolic processes to form inosine in the gut [35]. This metabolite is found in the highest concentrations within the duodenum of the small intestine, decreasing in the systemic concentration in the lower parts of the GIT [35]. Despite these fundamental roles, the biological properties of inosine are not well understood, and the exact targets of this compound within the body are poorly defined. Inosine has also not been well-researched as an antiproliferative agent preclinically, and further in vitro studies are warranted to decipher both the biological and antiproliferative potential of this metabolite in humans.

To date, limited research has been conducted into the anticancer properties and mechanisms of action of inosine against breast cancer. The potential application of inosine as an antiproliferative agent is based on the observation that the introduction of inosine-producing bacterial species can increase overall host immunity and the efficacy of immune checkpoint blockade therapies [35]. Our previous research assessed the antiproliferative action of inosine against the oestrogen-dependent MCF7 and triple-negative MDA-MB-231 human breast adenocarcinoma cell lines, in which the IC_50_ value of inosine was found to be 10.23 mM and 2.44 mM, respectively, indicating a potential affinitive for inosine to inhibit cell proliferation of the MDA-MB-231 cells above MCF7 cells [29]. Furthermore, inosine was found to inhibit the production of ROS by 62.94% and 65.03% at 11.18 mM and 7.46 mM, respectively, against the MDA-MB-231 TNBC cells [29]. This study also found that inosine was able to induce apoptotic cell death in both breast cancer cell lines at the maximum tested concentration of 11.18 mM, with statistical significance (*p* < 0.0001) observed compared to the negative control against MCF7 cells [29]. Notably, proteomic analysis of inosine-treated MCF7 cells revealed the potential induction of cell cycle arrest in the G_1_/S and G_2_M growth phases; however, this would need to be further validated with cell cycle-specific assays [29]. Significantly, previous research has acknowledged that inosine is biologically active under hypoxic conditions in breast cancer, and under select physiological conditions, inosine can serve as a more potent cytoprotective compound than adenosine, providing a novel insight into the immunosuppressive actions of this metabolite within the tumour microenvironment [36]. The physiological potential of inosine under normal and disease conditions warrants further investigation to ascertain the extent of these properties within the body and the associated mechanisms of action.

#### 3.1.3. Ellagic Acid Derivatives

Ellagic acid (EA) and ellagitannins are produced as a result of the metabolic breakdown of punicalagin in the intestine, which is a bioactive hydrolysable polyphenol group present in pomegranate [37], and have also been found in abundance in raspberries, chestnuts, strawberries, tea, walnuts, mulberries, ground elm, and blueberries [38]. Ellagitannins have been associated with the alleviation of adverse effects of metabolic disorders and diabetes; however, they have limited bioavailability and cannot reach systemic circulation in significant concentrations [38]. Ellagitannins have been found in higher concentrations within the GIT because they are metabolised into EA in the colon, stomach, and small intestine; however, EA is poorly absorbed from the GIT, which reduces the bioavailability of this compound in the host [38]. Nonetheless, when ellagitannins are hydrolysed to produce EA, they are able to reach the colon to be further hydrolysed and metabolised by gut microbial species into urolithins [38]. These urolithin metabolites, including urolithin A and urolithin B, have been observed to possess significant antioxidant activity and can be useful in formulating evidence-based therapeutic approaches [37,39]. In addition to their antioxidant potential, urolithin A and B have also demonstrated antiproliferative, anti-inflammatory, and estrogenic properties; however, the extent by which urolithin A, as the most active and effective urolithin, can impact the function of immune cells remains speculative. A preclinical study aimed to examine the action of urolithin A on immune cell activity, and it was observed that urolithin A was able to upregulate levels of miR-10a-5p, downregulating expression of Orai1/STIM1/STIM2, modulating Ca^2+^ entry into CD4^+^ T cells, and effectively inhibiting the proliferation and activation of murine CD4^+^ T cells [40]. Through these observations, an identifiable correlation between the upregulation of miR-10a-5p and the suppression of CD4^+^ T cells could be established, which is significant in understanding the mechanisms by which urolithin A exerts its effects [40]. These findings supported the potential use of urolithin A as a natural immunosuppressant in the treatment of inflammatory disorders, such as inflammatory bowel disease, and the mitigation of associated adverse events.

The capacity of urolithins to influence the immune response and exert antioxidant activity supports their potential use as antiproliferative agents against breast cancer. Studies on EA derivatives have primarily investigated the application of urolithin A as an antiproliferative agent, as it is the most bioactive urolithin with significant anti-inflammatory and antioxidant properties against breast cancer cells. EA-derived urolithin A and its sulphate conjugate were found to be substrates for the drug efflux transporter breast cancer resistance protein (ABCG2/BCRP), which impacts the pharmacological properties of various molecules [41]. These findings identified that physiologically bioavailable concentrations of EA-derived urolithin A could regulate ABCG2/BCRP-mediated transport and influence mechanisms of cancer drug resistance [41]. One recent study utilised molecular docking to examine the biological properties of urolithin A and found that it reduced breast cancer cell viability dose-dependently with an IC_50_ value of 443 µg/mL against the MDA-MB-231 TNBC cells [42].

Following consumption of urolithin A, in vivo hollow fibre assay results demonstrated reduced cell viability in breast cancer cells, and it did not alter blood biochemical parameters or induce uterine proliferation [43]. These findings also identified that urolithin A has a well-tolerated safety profile for consumption [43]. A recently published study aimed to investigate the mechanisms by which urolithin A regulated tumour macrophages and tumour cells in the inhibition of breast cancer progression [44]. Treatment with urolithin A inhibited two harmful inflammatory factors, TNF-α and IL-6, in triple-negative breast cancer cell lines and tumour-associated macrophages, attenuating tumour progression and alleviating the tumour microenvironment [44]. This compound facilitated the clearance of damaged mitochondria, which reduced the release of toxic inflammatory factors from damaged mitochondria [44]. As a crucial downstream target in tumour macrophages, the observed action of urolithin A against TFEB in multiple breast cancer models provides a novel understanding of the mechanisms by which this compound could act as an anticancer drug. However, the biological properties and mechanisms of action of other common EA-derived metabolites, such as urolithin B, have not been thoroughly studied against breast cancer.

#### 3.1.4. Tryptophan Derivatives

Tryptophan has been well-researched for its biological properties as an essential amino acid used in the biosynthesis of proteins, as it is a biosynthetic precursor to various gut metabolites [45]. The most common natural food sources of tryptophan are milk, meat, eggs, chocolate, oats, bread, and peanuts [45,46]. Additionally, some gut bacterial species, such as *E. coli*, are able to produce bacterial-derived tryptophan within the host [45,46]. The resulting metabolites of the metabolic conversion of tryptophan have a significant role in signalling pathways within the microbial community and serve as a connection for host-microbe interactions to maintain gut homeostasis within the host [46]. Approximately 4–6% of tryptophan is metabolised into indole derivatives (e.g., indole propionic acid, indole acetic acid, tryptamine, indole, and skatole) via bacterial enzymes, such as tryptophanase, and these molecules can function to enhance gut barrier function and modulate immune system responses within the body [46,47]. Indole acetic acid and indole propionic acid are two key tryptophan metabolites that have been observed to play a role in host immunity and intestinal permeability [45]. Additionally, indole has been identified as an interspecies signalling molecule, as it is able to control physiological processes, including antibiotic resistance, biofilm formation, and sporulation; however, the specific microbial enzymatic pathways involved in the production and bioactivity of indole are not well understood [45].

The correlation between tryptophan and cancer progression has been investigated previously as a potential tool to screen people with cancer. One study examined the plasma samples of participants with colorectal, gastric, lung, esophageal, and breast cancers, as well as that of healthy controls, and observed that tryptophan was altered similarly in all participants with cancer [48]. In these cancer types, metabolic pathway analysis identified tryptophan metabolism as one of the most significant enrichment pathways, supporting the potential role of tryptophan levels in the pathogenesis of people living with cancer [48]. It has also been observed that high extracellular levels of tryptophan are associated with poorer survival in participants with breast cancer [49]; however, a subsequent study identified a potential protective role of indole in breast cancer survival, with indole levels observed to be downregulated in progressive cases of breast cancer [50].

Indole derivatives have been observed to inhibit carcinogenesis via the maintenance of gut homeostasis, such as by upregulating tight junctions, cell turnover, AMP secretion, and mucin. Indole propionic acid, specifically, has exhibited potent antioxidant activity as a free radical scavenger, preventing DNA damage in non-transformed cells exposed to different types of oxidative stress [51]. Indole propionic acid was found to be selective for breast cancer cells, and not fibroblasts, and in vitro and in vivo supplementation with this metabolite reduced the proliferation and metastatic progression of breast cancer cells, as well as reducing the abundance of cancer stem cells [47]. The cytoprotective potential of indole propionic acid was found to be due to the downregulation of NRF2 (nuclear factor erythroid 2-related factor 2), enhanced production of mitochondrial reactive species, and the upregulation of iNOS (inducible nitric oxide synthase), which collectively induced oxidative and nitrosative stress by inhibiting EMT (epithelial-to-mesenchymal transition) [47]. Indole propionic exerted its cytoprotective effects via the PXR (pregnane X receptor) and AHR (aryl hydrocarbon receptor) receptors, and an increase in PXR and AHR levels was associated with better survival in participants with breast cancer [47]. The expression of PXR and activation of AHR was observed to be inversely related to the stage and grade of the breast tumour, and in women newly diagnosed with breast cancer, indole propionic acid synthesis within the faecal microbiome was suppressed [47]. Additionally, studies have observed that indole propionic acid exerted its cytoprotective effects in TNBC cells by reducing cancer cell stemness, as well as their EMT and proliferation [52]. Among the studied bacterial metabolites, indole propionic acid and cadaverine were found to induce greater levels of tumour-infiltrating lymphocytes and reduce the abundance of ALDH1+ cancer stem cells in cultured cells, which contributed to a reduction in recurrence and therapy resistance [47,53].

Metagenomic analyses observed different gut microbial signatures between neoadjuvant chemotherapy (NAC) effectual and non-effectual participants with breast cancer groups [54]. The gut microbiota of the NAC non-effectual group presented with low diversity and abundance of microbial communities, with a distinct reduction in indole propionic acid-producing and butyrate-producing bacteria and an increase in potential pathogenic microbes, in comparison to the NAC effectual group [54]. As the production of indole propionic acid was found to be suppressed in participants with breast cancer, this metabolite could play a role in the management of breast cancer. Whilst intestinal indole-mediated activation of AHRs has exhibited suppressive effects on inflammatory processes and colon carcinogenesis, there has also been evidence to support the role of AHR activation in tumour development and mutagenesis in other regions of the body [6].

### 3.2. The Association Between the Gut Microbiota and Standard Chemotherapeutic Drugs

The positive impact of the gut microbiota on standard chemotherapeutics has been observed in preclinical research in recent years. Current chemotherapeutics possess clinical limitations that reduce their overall efficacy, including poor bioavailability, non-selective distribution, rapid blood clearance, and the development of drug resistance [55]. Gut microbial species have been found to modulate the efficacy and toxicity of several standard chemotherapeutic agents, including CTX, 5-Fluorouracil, cisplatin, gemcitabine, and irinotecan, as well as hormone-, protein-, and enzyme-specific targeted therapies, including tyrosine kinase inhibitors [56]. In addition, the gut microbial composition has also been found to affect the efficacy and toxicity of cancer immunotherapies [56]. In a bidirectional manner, gut microbiota manipulation could improve overall chemotherapy efficacy, and their toxicity could be reduced, through pre-, pro-, and postbiotics, or faecal transplantation [56].

The gut microbiota has been reported to influence both the efficacy and toxicity of NAC, which alters the host response to the therapy [24]. For example, doxorubicin and CTX initiate the translocation of specific gut commensal bacterial species, including *Lactobacillus johnsonii*, *Enterococcus hirae*, and *Barnesiella intestinihominis*, into secondary lymphoid organs [24]. These gut bacterial species can reduce immunosuppressive intratumoural T-regulators, which reduce tumour growth as a causative effect of chemotherapy [24]. The modulation of host CD4^+^ T lymphocytes by the gut microbial species may be the underlying mechanism of action driving chemosensitivity and NAC pathologic response, in which gut microbes can serve as potential biomarkers for NAC patient response and a novel intervention target for NAC non-effectual breast cancer patients [54].

One study implemented shotgun metagenomics sequencing on faecal samples of early-stage breast cancer patients at diagnosis and following adjuvant chemotherapy and observed that distinct commensal bacterial species residing in the gut impact breast cancer prognosis in patients and breast cancer tumour aggressiveness in mice [57]. However, this study also acknowledged that chemotherapy could disrupt the healthy microbial composition for unfavourable pathogenic species, and selected commensal species can impact the probability of developing neurological side effects [57]. Significantly, these findings have acknowledged that the abundance of healthy or toxic commensal species in the gut may be able to predict the therapeutic efficacy of adjuvant or neoadjuvant chemotherapy [57].

The use of gut microbial signatures for the prediction of therapeutic efficacy could play a crucial role in mitigating the poor prognoses associated with TNBC in the early stages. Evidence suggests that introducing gut microbial metabolites, such as SCFAs (e.g., sodium butyrate), into cancer treatment regimens alleviated adverse events associated with standard chemo- and radiotherapeutic treatments in participants with cancer [58]. Alternatively, the metabolic effects of some microbial metabolites on specific anticancer drugs can render the drugs ineffective or increase their adverse effects, depending on the type of cancer [58]. Administering cisplatin as a chemotherapeutic has been associated with significant adverse events, including intestinal toxicity and weight loss, which impacts its efficacy and far-reaching effects within the host, and oral supplementation of *Lactobacillus* has been found to reduce the toxic side effects associated with cisplatin application in mice [55]. The therapeutic benefits associated with combining different treatment therapies in the management of breast cancer are also illustrated, with some combined approaches producing synergistic activity in limiting cancer progression [4]. Many studies on the treatment of TNBC have combined HDACi with standard anticancer approaches, including kinase and autophagy inhibitors, antibiotics, chemotherapy, or the combination of HDACi that display synergistic potential [4]. In many settings, the combined approach of HDACi and another compound, including kinase inhibitors, ionising radiation, autophagy inhibitors, or two HDAC inhibitors together, increased tumour growth inhibition through antiproliferative effects and prevented metastasis [4].

A bidirectional influence between the gut microbiota and standard chemotherapeutic drugs also exists. Whilst gut microbial metabolites have been found to increase the therapeutic efficacy and reduce the adverse events of chemotherapy drugs [58], standard chemotherapeutics can also disrupt the healthy gut microbial composition and trigger inappropriate immune responses within the gut, such as inflammatory processes [24]. Chemotherapeutic drugs, such as 5-fluorouracil and CTX, are toxic to the healthy gut microbial communities and act directly to disrupt the standard composition of the gut microbiota or induce inappropriate immune responses to target these microbial species [24]. To examine these changes, a pilot longitudinal study investigated alterations to the alpha diversity and bacterial species abundance in participants with breast cancer in the first year of treatment following chemotherapy treatment, in which all treatment types were found to impact the patient gut microbiota [25]. Specifically, there was an observed variation in *Firmicutes*, *Bacteroidetes*, and *Verrucomicrobia* taxa and alpha diversity abundance across the different treatment groups in this clinical study [25]. A cross-sectional study explored the correlation between gut microbial diversity and composition in women diagnosed with breast cancer and undergoing chemotherapy treatment in comparison to cancer-free healthy controls [59]. This study identified a prevalent difference in gut microbial composition between breast cancer patients and healthy controls, characterised by a reduced abundance of the mucin-degrading genus *Akkermansia* in participants with breast cancer compared to controls [59]. Collectively, these findings supported that gut microbiota-based interventions may improve quality of life outcomes in participants with breast cancer.

Emerging evidence also suggests that NOD1 (nucleotide-binding oligomerisation domain-containing protein 1), an intracellular pattern recognition receptor, possesses multifaceted contributions to cancer progression. Activation of NOD1 triggers notable signalling pathways (such as NF-kB and MAPK) that lead to inflammatory responses and production of cytokines, as well as influencing mechanistic processes, such as apoptosis, stemness, and chemoresistance [60]. One study observed that gut dysbiosis can induce psychological stress-triggered cancer stemness via activation of the LRP5-β-catenin pathway [61]. Metabolomic and metagenomic analyses of the gut microbial communities observed that the most significant alterations were a reduced abundance in *Akkermansia muciniphila* species, as well as a decrease in SCFA butyrate and overall composition and abundance of gut microbiota [61]. This suggested the potential use of the gut microbiota as a therapeutic approach for people with cancer experiencing psychological stress, as well as the identification of clinical biomarkers [61]. Furthermore, beneficial (*Faecalibacterium prausnitzii*, *Eubacterium rectale*/*Roseburia*, and *Firmicutes*) or harmful (*Clostridiales* and *Escherichia coli*) gut microbial species serve as a potential link between the gut and tumour microbiome, as well as a response to standard anticancer therapies [62]. Recent studies have highlighted the potential of microbiota modulation, via probiotic supplementation or faecal microbiota transplantation, to overcome chemoresistance observed with standard chemotherapeutic drugs [62]. In specific relation to breast cancer, overexpression of NOD1 was found to reduce cell proliferation in the highly invasive Hs578T breast cancer cell line, as well as increase clonogenic potential in vitro [63]. To date, in vivo and clinical research have not investigated the potential role of NOD1 in breast cancer. These findings emphasised the potential of gut microbial-derived signals interacting with NOD1 to influence therapeutic outcomes in breast cancer, warranting further investigation into this emerging aspect of gut microbiota and oncology.

### 3.3. The Gut Microbiota and Epigenetic Modification

#### 3.3.1. The Gut Microbiota-microRNA Connection

The gut microbiota-microRNA (miRNA) connection is integral in understanding the molecular pathways involved in mediating the diverse functions of the gut microbiota in the body. miRNAs are small single-stranded non-coding RNA molecules and are involved in various physiological processes via the regulation of gene expression [64,65]. Mammalian miRNAs are responsible for the regulation of roughly 30% of all protein-coding genes, and the complex regulatory network derived from this activity elucidates the significant role of miRNAs in normal physiological functions, such as immunity [64]. These epigenetic molecules can contribute to both adaptive and innate immunity due to their capacity to control the differentiation and function of various immune cell types [66]. The correlation between miRNA expression and immune-related disorders has been well-researched [67], however research on the role of miRNAs within the intestinal immune system is limited.

MiRNA expression has been associated with the maintenance of gut homeostasis as it has been observed to play a potential role in how microbiota-induced signals are received and prevent inappropriate inflammation in response to gut microbial species [67]. It has also been observed that gut commensal bacterial species play a vital role in the development and modulation of the host intestinal immune system, and emerging research has acknowledged that miRNA-induced gene silencing is involved in this regulation [64]. The gut microbial species communicate with host miRNA expression through gut microbial metabolites and lipopolysaccharides, whereby these signalling messengers travel in the systemic circulation to metabolic organs and regulate gene expression via miRNA interference [68]. As a bidirectional connection, gut metabolites have been observed to influence intestinal miRNA expression, and host-derived miRNAs secreted into the intestinal lumen have also been found to alter gut microbial composition and physiological function, including maintaining intestinal barrier integrity [64].

In the same manner that miRNA expression can maintain gut homeostasis, gut dysbiosis can trigger disturbances to standard physiological miRNA expression within the host. Understanding the underlying mechanisms between gut microbiota and miRNA interactions may be instrumental in developing novel strategies for treating metabolic disorders. In a clinical context, extracellular vesicle-derived miRNAs serve as a novel regulatory network for the GBA and may present as therapeutic targets in the modulation of the GBA for various disease conditions [69].

#### 3.3.2. Prevalent miRNAs Associated with the Gut Microbiota as Potential Epigenetic Therapeutic Targets

Understanding the miRNAs associated with the gut microbiota is vital in identifying potential epigenetic therapeutic targets in the maintenance of health and prevention of diseases. Of specific importance to the modulation of immune system processes, key miRNAs that have been most well-studied include miR-155, miR-146a, miRs-17∼92, and miR-181a [67]. These miRNAs regulate critical signalling pathways within the host, including NF-κB, TLR/MyD88, Jak/Stat, and Akt, which are integral to the immune responses [67]. In specific relation to the intestinal immune system, studied miRNAs that have an observed effect or role within the intestinal system include miR-155, miR-29, miR-10a, miR-146a, miR-122, miR-124, miR-21, and miR-143/miR-145, with diverse properties including anti-inflammation and intestinal epithelial regeneration [67]. Notably, miR-10a is one of the most abundant anti-inflammatory miRNAs that has been observed to be associated with various inflammatory disease conditions, including inflammatory bowel disease (IBD), rheumatoid arthritis, colitis, atherosclerosis, acute pancreatitis, sepsis, and cancer [70]. MiR-10a is a post-transcriptional mediator in controlling inflammatory responses within the host, and the actions of this molecule are well-conserved among vertebrates in general [70]. The gut microbial species have been found to negatively regulate intestinal miR-10a expression in vivo, in which intestinal cells of specific pathogen-free mice exhibited lower miR-10a levels than those of germ-free mice [66]. In comparison to control mice, mice with colitis were observed to show higher levels of IL-12/IL-23p40 expression and lower levels of miR-10a expression, indicating that the regulation of gut homeostasis by gut microbial species may occur by targeting IL-12/IL-23p40 expression to negatively regulate miR-10a levels, as a potential therapeutic target [66].

Several miRNA molecules, including miR-200a, miR-152, miR-30a-5p, miR-181a, miR-210, miR-24, miR-148a, miR-27a, miR-26a, miR-29a, miR-27b, and miR-25, have been correlated to patients with type I diabetes [71]. Both miR-375 and miR-278 play an essential role in modulating insulin secretion. In particular, the overexpression of miR-375 inhibited glucose-induced insulin secretion, and this action was reversed when miR-375 expression was downregulated [71]. One in vivo study aimed to determine the effects of miR-10a-5p on insulin resistance, as well as the diurnal patterns of gut microbiota and serum triglycerides in high-fat diet-fed mice, and observed that the oral administration of miR-10a-5p (100 pmol/mice per day) substantially increased glucose tolerance and insulin sensitivity and prevented body weight gain in mice [72]. These biological properties of miR-10a-5p oral administration were determined to be due to the modulation of the *Lachnospiraceae* diurnal rhythm and the *Lachnospiraceae* metabolite butyrate [72]. Significantly, dysregulation of miRNA expression has been shown to play a vital role in cancer, with a clear differential miRNA expression between cancer tissue and normal tissue. Overexpression and dysregulation of miRNAs have been affiliated with liver, lung, colorectal, pancreatic, lymphoma, leukaemia, and breast malignancies [71].

### 3.4. The Gut Microbiota-miRNA-Breast Cancer Connection

#### 3.4.1. Prevalent miRNAs Associated with Breast Cancer as Potential Epigenetic Therapeutic Targets

**Figure 4:**
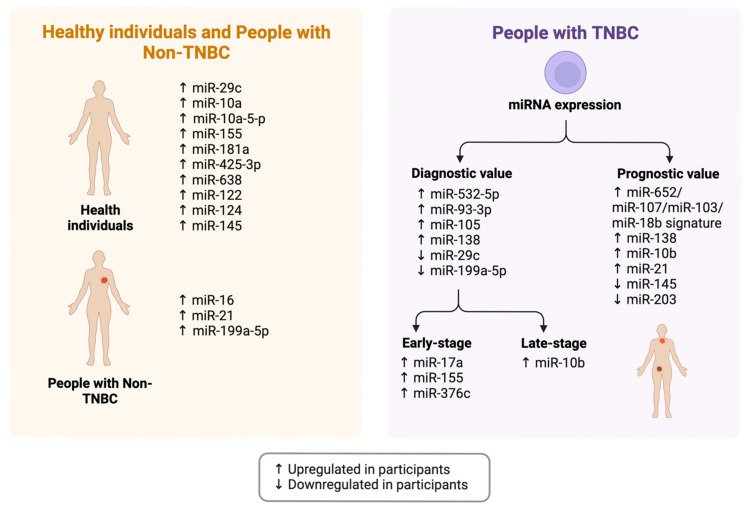
The up- and downregulation of key miRNA expression in healthy controls and people with non-TNBC and TNBC.

The identification of specific prognostic markers and epigenetic targets for TNBC tumour recurrence and metastasis may prove instrumental in the prediction of clinical outcomes of breast cancer (Figure 4). Stably expressed miRNAs in human serum have been found to predict patient prognosis with breast cancer, and the identification of these circulating biomarkers is minimally invasive, and it may lead to better-targeted treatment options for TNBC patients. Determining the molecular signature of the precancerous TNBC state will aid in the identification of subtype-specific biomarkers for breast cancer risk, and it will also contribute to the identification of molecular pathways that can be specifically targeted for subtype-specific prevention.

Using The Cancer Genome Atlas (TCGA) and model cell lines for breast cancer progression, one study investigated common miRNA signatures in TNBC as potential biomarkers, and miR-29c was identified as a lead candidate for early detection and targets for prevention [73]. MiR-29c was found to be upregulated in the normal breast epithelial MCF10A cell line and decreased steadily in the TNBC progression model, it significantly inhibited the cell proliferation of the MDA-MB-231 metastatic TNBC cell line, and it had several downstream targets for drug administration [73]. This includes the DNA methyl transferase DNMT3A, which inversely correlates to miR-29c and increases in expression in the progression from normal to cancerous state in a TNBC model [73]. Other downstream targets of miR-29c included src kinase, GSK-3β, Pan-ErbB TK, EGFR, MEK1/2, PI3K, PI3Kγ and c-met [73]. The observed connection between miR-29c and DNMT3A was indicative of a probable epigenetic component to tumorigenesis in TNBC, which has been further investigated in a subsequent study, and the authors suggested that the miR-29c network may play an essential role, and therapeutic target, in the development and progression of TNBC [73].

Another study performed genome-wide serum miRNA expression and real-time PCR analyses to predict tumour relapse in sixty primary TNBC patients by analysing miRNA expression, in which a four-miRNA signature (miR-652, miR-107, miR-103, and miR-18b) was found to be a predictor of TNBC tumour relapse and overall survival [74]. Based on these findings, multivariate Cox regression statistical models indicated that the high-risk score based on the four-miRNA signature was an independent prognostic indicator of TNBC patients [74]. The role of specific miRNA clusters and signatures in the biology of TNBC could strengthen clinical understanding of disease prognosis and therapeutic approach. A later study reported dysregulation of several miRNAs of prognostic value in TNBC patients, including miR-10b, miR-145, miR-21, miR-203, miR-29, miR-200 family, and miR-221/222 [75]. Additionally, it has been observed that miR-532-5p can be upregulated in TNBC patients [76].

Given the aggressive nature of the TNBC subtype, its early diagnosis should be prioritised for optimal disease management. The blood samples of TNBC patients and non-TNBC patients were collected to examine the differential expression of circulating miRNAs, in which miR-17a, miR-155, and miR-376c were identified as biomarkers of early-stage TNBC and miR-10b was found to be a late-stage TNBC biomarker [77]. Another study comparing miRNA expression in TNBC and non-TNBC patients observed that miR-16, miR-21, and miR-199a-5p were downregulated in TNBC compared to non-TNBC participants [78]. The data collated from the study considered miR-199a-5p a TNBC-specific biomarker with diagnostic value, which could provide insights into using miR-199a-5p as a target for future TNBC drug discovery [78].

It has been acknowledged that increased miR-138 expression is highly specific to the TNBC subtype and functionally relevant to breast cancer progression, and it correlates with poor prognosis in TNBC. One study investigated the aberrant expression of miR-138 in TNBC to determine its role in tumorigenesis and established miR-138 as both a specific diagnostic and prognostic marker for TNBC [79]. This miRNA directly targets specific tumour-suppressive and pro-apoptotic genes, including the silencing of *TUSC2* (tumour suppressor candidate 2) [79]. Overexpression of *TUSC2* mimics miR-138 knockdown, and this gene serves as a direct downstream target of miR-138, as validated by functional rescue experiments [79]. The observation of miR-138 as an oncogenic promoter in TNBC indicates its use as a target for oligonucleotide therapeutics, with the potential use of anti-miR-138 as an adjuvant therapy in TNBC. One study observed differential miRNA expressions across age groups, identifying that in very young participants with breast cancer, evolutionarily conserved miRNA clusters were upregulated, which is responsible for the initiation of tumorigenesis [76]. Whilst it is well-established that the TNBC subtype has a poor prognosis, the additional risk factor of age should be considered when evaluating the predictive potential of miRNA expression in TNBC.

As a significant factor of consideration for patient response to treatment, dysregulation of miRNA expression has also been associated with chemoresistance in TNBC. The miRNA array profiles of breast cancer patients observed that miR-93-3p and miR-105 expressions were upregulated in TNBC and correlated with poor survival [80]. Both miRNAs were found to activate Wnt/ß-catenin signalling via the downregulation of SFPR1, which effectively promoted stemness, metastasis, and, most notably, chemoresistance [80]. The combined circulating expression of miR-105/miR-93-3p warrants further development into its use as a diagnostic biomarker in early- and late-stage TNBC [80]. Additionally, elevated miR-181a expression has correlated with non-responsiveness to NAC, and reduced expression of miR-200c has shown chemoresistance and poor response to radiotherapy [81]. Identifying epigenetic targets that may contribute to patient therapeutic response is vital in combatting the aggressive nature of TNBC.

#### 3.4.2. The Association Between the Gut Microbiota, miRNAs, and Breast Cancer

The literature does not well characterise the interplay between gut microbial metabolites, miRNA expression, and breast cancer progression. However, the identifiable association between miRNA expression and TNBC prognosis and the gut microbiota’s influence on the alteration of miRNA expression indicate a possible link in understanding the mechanisms of action of gut metabolites against breast cancer.

As sodium butyrate was observed to possess promising antiproliferative potential against breast cancer cells, one study investigated the effect of this metabolite on the expression of miR-101 in the MDA-MB-468 TNBC cell line [82]. The study implemented scratch and transwell assay and real-time polymerase chain reaction (PCR) to measure gene expression in the TNBC metastatic process [82]. The findings of this study indicated that sodium butyrate upregulated tumour suppressor miR-101 expression in the MDA-MB-468 cells, which confirmed its potency as an antiproliferative drug with anti-metastatic potential [82]. The significance of these findings supported the use of sodium butyrate as an anticancer drug in inhibiting migration and invasion of TNBC cells. However, future research would need to investigate the mechanism behind the anti-metastatic observations.

To investigate the sensitivity of the MDA-MB-435 TNBC cell line to paclitaxel, a standard chemotherapeutic drug used to treat different cancers, the functional role of both miR-101 and MCL-1 (myeloid cell leukaemia 1) was investigated, as MCL-1 plays a key role in apoptosis [83]. The study found a negative correlation between miR-101 and MCL-1 expression in TNBC; specifically, miR-101 was significantly downregulated in TNBC tissues and cell lines, with strong expression of MCL-1 [83]. Of particular note, miR-101 increased paclitaxel sensitivity in the MDA-MB-435 cells by inhibiting MCL-1 expression [83]. An earlier study investigated the combined activity of sodium butyrate and chemotherapy (VP-16) on Burkitt lymphoma, an aggressive B-cell lymphoma more common in children [84]. This study observed that the combined drug treatment increased miR-101 expression, along with miR-143 and miR-145 expression, in the Burkitt lymphoma cell line, in which the expression of these molecules is typically downregulated in Burkitt lymphoma patients [84]. This study highlighted the potential use of sodium butyrate in combination with standard chemotherapy to improve the clinical outcome of cancer in the future and further validates previous observations that sodium butyrate acted on miR-101 expression within the tumour microenvironment [82,84]. Modulating miR-101 expression by sodium butyrate in different cancer types serves as a potential epigenetic target in inhibiting TNBC progression, as miR-101 expression is downregulated in TNBC cell lines, and could improve anticancer drug potency.

Several specific miRNAs can function as either oncogenes (oncomiRs) or tumour-suppressive miRs, and these molecules are often dysregulated in cancer cells. For example, the miR-31 is found to be overexpressed in metastatic breast cancer and it promotes several oncogenic processes, including invasion, proliferation, and motility. This miRNA has been identified as a novel target for HDACi, such as sodium butyrate, in breast cancer cells [85]. Sodium butyrate, a broad-spectrum HDACi, has been observed to upregulate miR-31 and induce cellular senescence in MDA-MB-231 TNBC cells [85]. Furthermore, inhibition of miR-31 expression overrode the HDACi-mediated induction of senescence, indicating that miR-31 may be an important target of HDACi and is a significant regulator of breast cancer senescence [85].

Interestingly, the miR-155 molecule has been observed to play a significant role in the modulation of the intestinal immune system [67]. Increased expression of miR-155 has also been associated with an overall better survival of people with TNBCs, with miR-155 expression being identified as a potential prognostic marker in the use of ionising radiation (IR)-based therapeutic approaches [86]. The significance of miR-155 in both the intestinal immune system and TNBC tumour microenvironment serves as a possible therapeutic target for gut microbial metabolites, which may act on miR-155 directly or indirectly. Understanding the mechanisms by which standard chemotherapeutic drugs currently used in the treatment of breast cancer can modulate epigenetic targets is crucial to defining effective combination therapies of gut microbial metabolites and standard drugs to treat TNBC.

The relationship between the intratumoural microbiota and miRNA expression on breast cancer metastasis has been largely unexplored and could provide novel insights into new drug targets for metastatic breast cancer. A recent study employed qPCR and 16S RNA sequencing methodologies to examine the correlation between intratumoural microbial composition and miRNA expression on the metastatic development in breast cancer patients and observed an increase in expression of miR-342-5p, miR-149-5p, and miR-20b-5p [87]. Specifically, the metastatic breast cancer patients exhibited a greater abundance of pathogenic and pro-inflammatory species *Streptococcus epidermis*, *Corynebacterium kroppenstedtii*, *Haemophilus influenzae*, and *Corynebacterium aurimucosum* [87]. In contrast, the non-metastatic group presented with an abundance of probiotic species, including *Faecalibacterium prausnitzii*, *Parabacteroides distasonis*, *Lactobacillus iners*, and *Blautia obeum*, which collectively indicate a dynamic relationship between breast cancer metastasis and specific intratumoural bacterial taxa [87]. In normal breast tissue, the most abundant bacterial families are *Lactobacillaceae* (*Firmicutes* phylum), *Xanthomonadaceae* and *Acetobacterraceae* (*Proteobacteria* phylum) [88]. Molecular pathway analysis identified bacterial invasion of epithelial cells, cell signalling, transcriptional activity, and extracellular matrix remodelling pathways upregulated in the metastatic group, highlighting potential roles of intratumoural bacterial taxa in breast cancer metastasis and progression [87]. Overarchingly, the observed perturbations in miRNA expression and species-level microbial abundance indicated a probable role in breast cancer metastatic progression and served as a strong prognostic signature for metastatic breast cancer [87]. The clinical relevance of this metastatic prognostic signature was validated in the observation that a low value of the signature was associated with higher overall survival in breast cancer patients, which supported the incorporation of intratumoural microbial abundance and miRNA expression in clinical risk assessments of breast cancer [87]. To improve patient outcomes and develop more targeted treatment strategies for advanced-stage and aggressive breast cancers, a collective consideration should be made for the gut microbiota, intertumoral composition, and miRNA expression.

### 3.5. Additional Diagnostic and Epigenetic Factors in the Management of Disease

#### Minimal Residual Disease (MRD)

MRD is a crucial factor of consideration in cancer therapy as it correlates with disease progression and clinical outcome. MRD encapsulates circulating tumour cells (CTCs), disseminated tumour cells (DTCs), and resistant cancer cells, which have survived initial tumour therapy and may contribute to metastasis or recurrence [89]. Emerging technologies aim to better understand the metastasis of carcinomas by interrelating the persistence of MRD, physiological properties of MRD, and overall patient prognosis [89]. The successful isolation and analysis of CTCs and DTCs is instrumental in determining the association between MRD and patient prognosis.

To ascertain the prognostic potential of MRD in women with early-stage breast cancer, a study aimed to determine the correlation between the presence of CTCs and DTCs with standard prognostic indicators in these patients [90]. The authors identified that CTCs were present in 31% of patients and DTCs were observed in 27% of patients, with no significant correlation between CTC or DTC occurrence and tumour classification, positive hormone status, and tumour histologic grade, indicating that CTCs and DTCs were not prognostic indicators in this study [90]. As the presence of CTCs and DTCs in early-stage breast cancer patients did not correlate with the standard breast cancer prognostic indicators, large-scale studies are required to assess their occurrence in early-stage breast cancer further [90].

The development of a large-scale sensitive MRD detection approach is crucial for the potential implementation of MRD detection in patients with metastatic breast cancer. Existing methodologies, such as cell-free DNA approaches, lack the high level of sensitivity required for detecting MRD post-therapy [91]. Given this limitation, researchers developed a test to track hundreds of patient-specific mutations to detect MRD in breast cancer with a 1000-fold lower error rate than conventional sequencing methods. Using this methodology, researchers compared the sensitivity of their approach to digital droplet PCR and retrospectively examined two patient cohorts- patients with metastatic breast cancer who were sampled six months post-metastatic diagnosis and patients with stage 0 to III breast cancer who received curative-intent treatment [91]. The present approach presented with clinical sensitivity at 81% in newly diagnosed metastatic breast cancer, 23% in postoperative patients, and 19% at one year in early-stage disease, and sensitivity was highest in patients with the greatest number of mutations that could be tracked [91]. Notably, the detection of MRD at one year was highly correlated with distant recurrence of cancer [91]. Significantly, the study quantifiably determined the strong association between MRD detection and breast cancer recurrence. However, the sensitivity of this approach was determined by the number of tumour mutations available to be tracked. For the successful incorporation of MRD detection in the clinical risk assessment of breast cancer, several areas should first be addressed, including further research for the characterisation of molecular and phenotypic features of MRD, evidence of patient care benefits in relation to therapeutic management via MRD identification, the establishment of a globally standardised approach for the isolation and identification of CTCs and DTCs, and a rapid and reliable MRD expansion method [89].

## 4. Conclusions and Future Directions

In recent years, a multifaceted link between gut microbial composition and breast cancer development has been observed. The bidirectional association between the gut microbiota and host health has widespread effects on the body, ranging from the primary target site to the distal regions of the body. The TNBC subtype is an aggressive form of breast cancer that has poor prognoses, a higher risk of metastasis, and limited treatment options. Therefore, novel and effective therapies are paramount to improving the clinical outcomes of TNBC. Identifying the specific molecular pathways involved in the antiproliferative activity of gut metabolites would give more insight into the mechanisms by which these metabolites can be utilised for therapeutic purposes. Specifically, investigating the bidirectional influence between gut microbial species and miRNA expression, both in healthy populations and in tumour microenvironments, will prove instrumental in understanding the epigenetic modulation involved in tumourigenesis. Research on the potential synergy between gut metabolites and standard chemotherapeutic drugs used in the treatment of breast cancer is limited, which could provide support for the development of gut microbiota-based combination therapies for breast cancer with reduced side effects. Clinical studies considering individual lifestyle factors and other compromising factors in defining gut microbiota-based therapies are crucial.

Current strategies implemented in microbiome research include large-scale metagenomics approaches, also referred to as shotgun sequencing, and a more targeted 16S rRNA gene next-generation sequencing approach [11]. Whilst current approaches have been useful in identifying potential molecular pathways involved in the antiproliferative action of gut microbial metabolites against breast cancer, more innovative methodologies are required to validate existing findings and conclusively determine these pathways. Examples of more innovative approaches include AI-based machine learning models for the personalisation of the gut microbial composition, targeted therapies (e.g., nanoparticle delivery) to minimise off-target effects and reduce potential toxicity, faecal microbial transplantation, lab-on-a-chip devices, metabolomics analysis for the evaluation of individual response to treatment, and microdevices to purify and detect circulating miRNAs. In addition to this, the use of multi-omics to profile microbiome–cancer interactions serves as a promising future direction for further developments within the field of oncological research. The genetic manipulation of genes involved in microbial metabolism can affect abundance of microbial metabolites using synthetic biology to alter the microbiome composition [58]. In the context of clinical practice, the integrative analysis of faecal and gut microbiome miRNA profiles may encourage the implementation of more accurate dietary patterns in the prevention and treatment of chronic conditions [92]. Whilst investigation into the potential therapeutic targets of gut metabolites and miRNA expression in the treatment of TNBC has demonstrated promising potential in improving patient care, further validation and development of more rapid and targeted methodologies is required.

To date, the potential application of gut microbial metabolites in adjuvant treatment for TNBC has limited clinical relevance and translation. The translation of preclinical (i.e., in vitro and in vivo) findings into clinical human studies faces several key issues, including bioavailability, pharmacokinetics, and the safety of administering gut metabolites in participants with TNBC. Clinical trials investigating the potential benefits associated with the administration of gut metabolites in cancer therapeutics or microbiome-based interventions are ongoing and will be of importance in the translation of preclinical findings into clinical practice.
